# The role of small adaptor proteins in the control of oncogenic signaling driven by tyrosine kinases in human cancer

**DOI:** 10.18632/oncotarget.6929

**Published:** 2016-01-17

**Authors:** Cécile Naudin, Clément Chevalier, Serge Roche

**Affiliations:** ^1^ CNRS UMR5237, University Montpellier, CRBM, Montpellier, France; ^2^ Present address: INSERM U1016, CNRS UMR8104, Institut Cochin, Paris, France; ^3^ Present address: SFR Biosit (UMS CNRS 3480/US INSERM 018), MRic Photonics Platform, University Rennes, Rennes, France; ^4^ Equipe Labellisée LIGUE 2014, Ligue Contre le Cancer, Paris, France

**Keywords:** cell signaling, tyrosine kinase, adaptor proteins, human cancer, cancer therapy

## Abstract

Protein phosphorylation on tyrosine (Tyr) residues has evolved as an important mechanism to coordinate cell communication in multicellular organisms. The importance of this process has been revealed by the discovery of the prominent oncogenic properties of tyrosine kinases (TK) upon deregulation of their physiological activities, often due to protein overexpression and/or somatic mutation. Recent reports suggest that TK oncogenic signaling is also under the control of small adaptor proteins. These cytosolic proteins lack intrinsic catalytic activity and signal by linking two functional members of a catalytic pathway. While most adaptors display positive regulatory functions, a small group of this family exerts negative regulatory functions by targeting several components of the TK signaling cascade. Here, we review how these less studied adaptor proteins negatively control TK activities and how their loss of function induces abnormal TK signaling, promoting tumor formation. We also discuss the therapeutic consequences of this novel regulatory mechanism in human oncology.

## INTRODUCTION

Protein phosphorylation on tyrosine residues catalyzed by tyrosine kinases (TKs) has evolved as an important mechanism to coordinate cell communication in multicellular organisms [1]. This molecular process is highly regulated *in vivo* because only less than 1% of mammalian proteins are phosphorylated on Tyr residues. Tyr phosphorylation (pTyr)-dependent signal transduction results from the combination of three molecular actions: initiation of the pTyr signal induced by the TK (the writer), propagation of the signal through recognition of the phosphorylated protein by a SRC Homology 2 (SH2) domain-containing protein (the reader) and control of the signal by phosphatases that dephosphorylate the substrate (the eraser) [1]. The human genome encodes about 90 TKs, 100 SH2 domain-containing proteins and 40 tyrosine phosphatases [2]. The tyrosine kinome consists of receptor and non-receptor TK families [3]. The Receptor TK (RTK) family includes receptors for growth factors and factors involved in cell adhesion and motility, cell survival and metabolism [4]. The non-receptor TK family comprises cytoplasmic TKs (CTKs) that mainly mediate signals transduced by receptors devoid of TK activity [5]. These non-enzymatic receptors can be activated by a large variety of extracellular cues, such as hormones, neurotransmitters, cytokines and antigens as well as components of the extracellular matrix, to regulate cell activity. Deregulation of this pTyr-dependent signaling has a strong effect in cancer [6]. Over 50% of TKs display aberrant activities in human tumors due to overexpression or somatic mutation of the corresponding gene and these molecular alterations are thought to be the main cause of oncogenic induction driven by abnormal pTyr-dependent protein phosphorylation in human cells [6]. Hence, small inhibitors or antibodies that target this molecular process have become an attractive therapeutic strategy in oncology [7]. However, TK inhibitors have shown variable effects in the clinic, suggesting that TK deregulation alone may not be always sufficient to induce oncogenesis and to predict tumor response to TK inhibitors [7].

In light to the complexity of TK signaling, this mechanism has been reported to also be negatively controlled by a class of “readers” composed of small adaptor proteins, as originally reported by Yoshimura *et al* [8]. Interestingly, inactivation of this regulatory mechanism has recently emerged as an additional important mechanism of oncogenic induction driven by aberrant TK activities. This review outlines the role of this class of adaptors in the control of pTyr-dependent signaling in normal and tumorigenic conditions and discusses the potential therapeutic implications of these novel findings.

## NEGATIVE REGULATION OF TK SIGNALING BY SMALL ADAPTORS IN NON-TRANSFORMED CELLS

Adaptor proteins define an important class of “readers” in the transmission of pTyr-dependent signaling. These proteins do not have enzymatic/catalytic or transcriptional activities, but act as molecular platforms that coordinate signaling events [9]. They mostly function as flexible molecular scaffolds that mediate protein–protein and protein–lipid interactions through interaction domains and binding motifs in their modular structure. These motifs allow specific interactions with effector proteins to regulate their localization and/or activities. Specifically, by binding to and bringing into proximity two or more signaling proteins, they can coordinate and regulate signaling events in space and time. Signaling proteins with “adaptor” function can be classified in three broad families: i) scaffold proteins that regulate a large number of effector proteins, ii) transmembrane proteins that dock signaling effectors at the plasma membrane, and iii) small cytoplasmic adaptors that bind two partners together [9]. This review will focus on this last class. The first small adaptors identified were either novel regulators of cell growth induced by growth factors or oncogenes (GRB2 and SHC) [10, 11], or transforming products of retroviruses (v-CRK) [12]. It is now established that this family of positive regulators includes members of the CRK, DAPP1, GRB2, NCK, SHB, SH3BP2, SHC, SH2D1-5, SLP76 and *STAP* families [2]. They can function through association with cognate effectors and subsequent targeting of the complex to the plasma membrane for activation. For example, in the cytoplasm, the adaptor GRB2 is constitutively associated with SOS, an activator of the small GTP-binding protein RAS, and upon growth factor stimulation, the complex is directed to the membrane by interaction via the SH2 domain with an RTK that enables RAS signaling [13].

**Figure 1:**
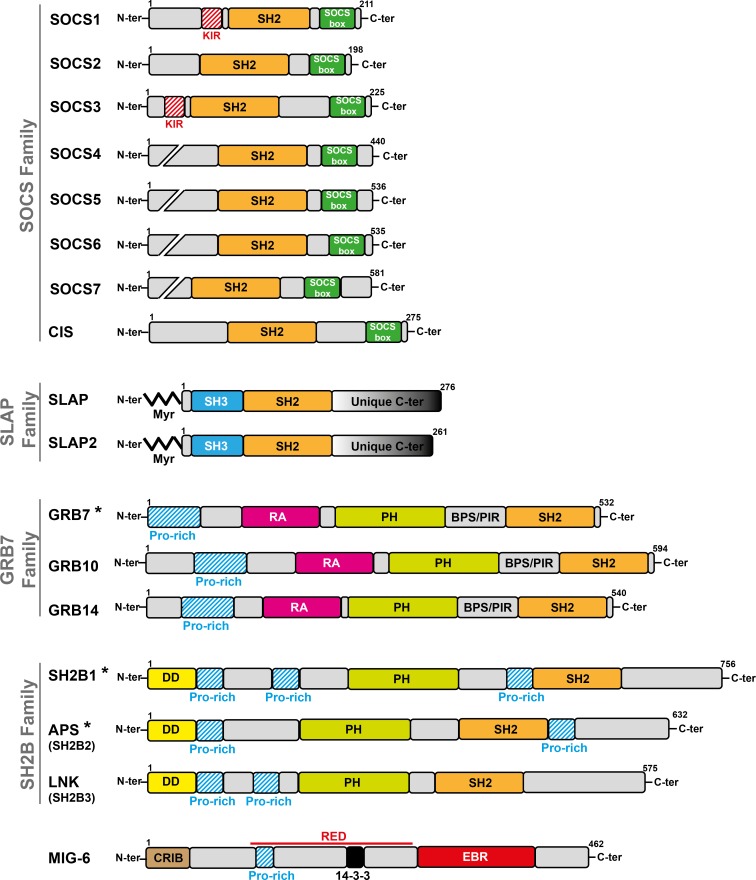
Modular structure of small adaptors that negatively regulate TK signaling The size of adaptor proteins (number of amino acids), presence of specific homology domains, sequences and myristoylation sites (Myr) are indicated. (*) indicates that the adaptor of this subfamily positively regulates TK signaling.

**Figure 2:**
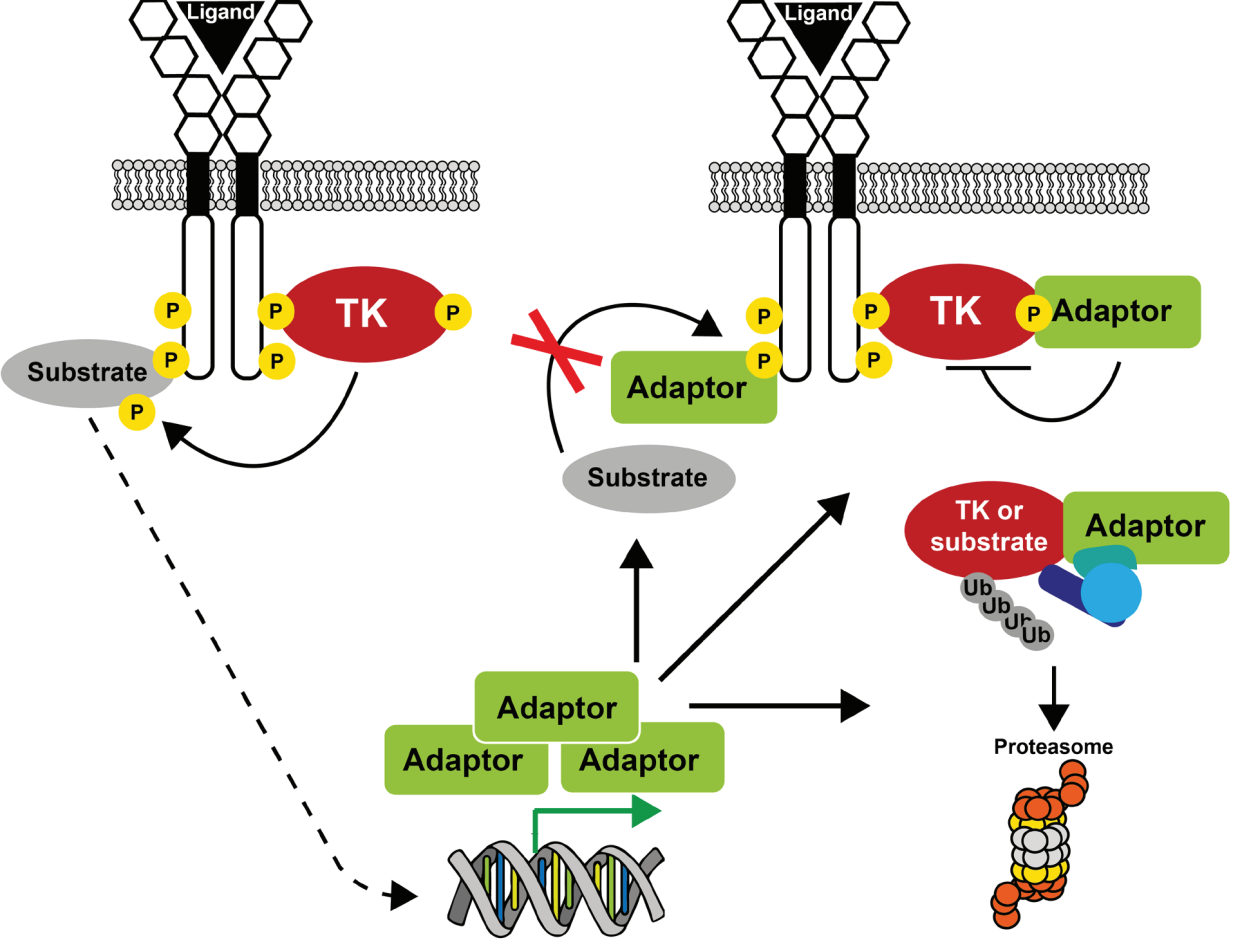
A unifying model on how small adaptors control TK signaling Sustained receptor stimulation generally induces expression of small adaptor proteins. As a result, the adaptor protein can inhibit TK signaling by competing with effectors/substrates for receptor binding, by directly inhibiting TK activity, or by promoting substrate/TK degradation via its association with a specific ubiquitination factor.

More recently, a new group of small adaptors has emerged that inhibits TK-induced cell responses. One of the first examples was cytokine inducible SH2-domain containing protein (CIS) [8] that was discovered while searching for new immediate early response genes induced by cytokines. CIS is a small molecule that contains an SH2 domain and has some homology with the transcription factor STAT5. When overexpressed, the cytokine response is inhibited. Since then, several adaptors with similar properties have been identified, among which members of the SOCS, SLAP, SH2B, GRB7 and MIG6 families are included (Figure 1). Except for SOCS family members, these adaptors have emerged late during evolution to finely tune signaling and to prevent unwanted cellular responses. They mostly define feed-back loops, as revealed by loss of function experiments in mice combined with biochemical studies in cultured cells. They can negatively regulate TK signaling through direct inhibition of the TK catalytic activity, competitive inhibition of TK association with downstream signaling proteins, or destabilization of specific components of the pTyr signaling cascade via the recruitment of ubiquitination factors (Figure 2). A detailed analysis of their role in TK signaling is presented below.

### SOCS family

The Suppressor of Cytokine Signaling (SOCS) family was originally identified based on the ability of its members to inhibit cytokine signaling and to bind to CTKs of the JAK family [14–16]. The mammalian SOCS family comprises eight members (SOCS1 to SOCS7 and CIS). They are composed of a variable N-terminal sequence, a central SH2 domain that shows homology with the STAT SH2, and a conserved sequence of about 40 amino acids, called “SOCS box” (Figure 1). This last domain is involved in the formation of Elongin/Cullin/SOCS box-type complexes that function as E3 ubiquitin ligases to promote the ubiquitination of targeted substrates. SOCS1 and SOCS3 additionally possess a short motif upstream of the SH2 domain that is called kinase-inhibitory region (KIR) and is critical for the inhibition of TK activity. SOCS proteins have emerged early in the animal kingdom. For instance, the *C. elegans* genome contains a single gene similar to *SOCS6* and *SOCS7*, while the *Drosophila* genome already contains three *SOCS* genes (*SOCS16D*, *SOCS36E* and *SOCS44A*) that are homologous to *SOCS4-7*. *Drosophila* SOCS proteins share similar functions with their human homologues. SOCS36E is both a target and a negative regulator of JAK/STAT signaling, while SOCS44A and SOCS36E are regulators of Epidermal Growth Factor (EGF) receptor signaling [17]. SOCS family members are expressed in a wide range of tissues with variations and specificities [18]. Cytokine and growth factor receptors induce *SOCS* gene expression, mostly via the STAT pathway, which in turn counteracts their signaling activity [19, 20].

Most SOCS family members can link the associated substrates to the ubiquitination machinery via the SOCS box. A wide range of substrates are targeted for proteolytic degradation, including CTKs of the JAK family, JAK-associated cytokine receptors, RTKs [19, 21], MAL (a component of the inflammatory toll-like receptor signaling cascade) and IRS1/2 (components of the metabolic insulin signaling pathway) [22, 23]. However, SOCS-mediated ubiquitination can also promote internalization and routing of receptors, for instance growth hormone and granulocyte-colony stimulating factor (G-CSF) receptors [24, 25]. This results in the receptor and, possibly, JAK turnover. SOCS proteins can also suppress signaling by competing with downstream signal transducers for binding to shared phosphorylated motifs of the activated receptors. Particularly, SOCS proteins block STAT recruitment to cytokine receptors by masking the STAT binding sites of such receptors [26]. Finally, only SOCS1 and SOCS3 can bind to JAKs via the SH2 domain and directly inhibit JAK catalytic activity via the KIR, which acts as a pseudo-substrate that impairs substrate accessibility [27, 28]. SOCS1 can directly bind to the phosphorylated Tyr1007 residue in JAK2 activation loop [29]. SOCS3 shows weak affinity for JAK, but binds to the cytokine receptor in close proximity of the kinase. Recent structural and mechanistic analyses have revealed the molecular basis of JAK inhibition by SOCS3 [30, 31]. Upon binding to JAKs, the unstructured KIR domain of SOCS3 adopts an extended β-strand-like conformation that sits in the catalytic pocket of the kinase, resulting in prevention of substrate binding or phosphorylation. SOCS3 can inhibit JAK1, JAK2 and TYK2 via its KIR, but not JAK3. This is due to the absence of an evolutionarily conserved Gly-Gln-Met sequence (GQM motif) in JAK3 kinase domain. SOCS3 interacts with this motif through its SH2 and KIR domains. To date, there are no structural data on SOCS1, but high sequence conservation in the KIR domain with SOCS3 suggests that it may inhibit JAKs through the same mechanism.

Genetically manipulated mice were used to determine the physiological functions of SOCS proteins and demonstrated that they have a crucial role in immunological processes and in growth control in accordance with *in vitro* observations (for review see [18]). For example, *Socs1*-deficient mice die within three weeks of birth due to severe systemic inflammation, resulting from uncontrolled interferon-γ (IFN-γ) signaling [32, 33]. Mice lacking *Socs3* die perinatally due to defective placental formation, whereas conditional *Socs3* depletion induces inflammatory and metabolic disorders [34–36]. Leukemia Inhibitory Factor Receptor (LIFR) gene deficiency was able to rescue the *Socs3* knockout placental defect and embryonic lethality, establishing SOCS3 as an essential regulator of LIFR signaling during placental formation [37]. Lethality of *Socs1* or *Socs3* deficient mice also revealed specific functions for these SOCS proteins that are not compensated by other family members. *Socs2*-deficient mice develop gigantism due to enhanced responses to growth hormone [38]. No *Cis* or *Socs4* knockout mouse model has been reported by now, but *Cis* transgenic mice exhibit growth retardation [39]. *Socs5* knockout mice showed no abnormalities, indicating possible redundancy between SOCS family members [40]. *Socs6* deficient mice displayed an 8-10% reduction in body weight, but, despite the *in vitro* data, *Socs6* knockout mice did not display any alterations in glucose metabolism [41]. Again, redundancy between SOCS family members may play a role in the absence of a phenotype in these mice. Finally, there have been conflicting reports regarding the *in vivo* function of SOCS7, probably due to differences in the genetic background of the respective mouse knockouts [42, 43]; however these observations suggested a role of SOCS7 in insulin signaling, consistent with the findings that SOCS7 can interact with the IR and their adaptor proteins.

### SLAP family

SRC-Like Adaptor Protein (SLAP) was identified by yeast two-hybrid genetic screening using the cytoplasmic domain of the RTK EPHA2 as bait [44]. SLAP2 was then discovered using bioinformatic and functional screening approaches [45–47]. SLAP displays considerable structural homology with SRC, but lacks the kinase domain. It has a unique myristoylated N terminus for membrane localization, followed by the SH3 and SH2 domains with high homology to those of SRC family TKs (about 50% identity) and a unique C terminus involved in the interaction with downstream signaling proteins, such as the ubiquitination factor CBL (Figure 1) [48]. SLAP proteins have emerged during vertebrate evolution by *SRC* duplication [2]. SLAP is strongly expressed in the hematopoietic system, epithelial intestine, lung and brain and more weakly in other tissues. SLAP2 expression is more specific to the hematopoietic tissue and lungs [48]. SLAP is implicated in the negative regulation of RTK and immunoreceptor signaling. Surprisingly, the expression of SLAP proteins does not seem to be induced by receptor stimulation and, thus, it may not define a negative-feedback loop requiring transcriptional activation and novel protein synthesis, as reported for SOCS proteins. Their expression is rather induced during cell differentiation, for instance during thymocyte development [49].

SLAP negatively regulates SRC signaling by targeting SRC substrates for degradation or by competing with SRC for association with upstream receptors. Due to the homology with the SRC SH2 domain, SLAP SH2 competes with SRC SH2 for platelet-derived growth factor (PDGF) receptor interaction and thereby impairs SRC-mediated mitogenic signaling [50]. However, SLAP does not share the SRC-binding of FLT3 or EPHA2 suggesting that this competitive mechanism does not operate for all RTKs [51, 52]. SLAP proteins are also implicated in the degradation of SRC-like signaling components by facilitating the recruitment of ubiquitin ligases. For instance, they inhibit T cell receptor (TCR) and B cell receptor (BCR) activities by docking CBL to components of these receptor complexes and inducing their degradation [45, 53–55]. This mechanism may require phosphorylation by the TK LCK and the SLAP SH2 domain [53]. SLAP SH3 domain must also be intact for optimal attenuation of TCR signaling [56]. A recent structural analysis of SLAP2 revealed that the SH3 and SH2 domains directly interact through a beta-sheet formation and this may be important for SH2 binding activity [57]. Due to the high identity between SLAP proteins, this mechanism may be also operative in SLAP and could explain, at least in part, the important role of SLAP SH3 in TCR signaling. In addition to its role in lymphocytes, SLAP also controls F-actin assembly induced by PDGF in a CBL-dependent manner in fibroblasts, probably through destabilization of an upstream regulator of RAC GTPases [58]. Finally, SLAP also participates in CBL-dependent ubiquitination, internalization and/or degradation of many receptors, including GM-CSF receptor [59], FLT3 [51], KIT [60] and colony stimulating factor 1 (CSF-1) receptor [61]. SLAP also interacts with erythropoietin (EPO) receptor and negatively regulates erythroid terminal differentiation by unknown mechanisms [62].

Several *in vivo* SLAP functions have been revealed by *Slap1* and *2* knockout experiments in mice (for reviews, see [48, 64, 65]). Surprisingly, these animals are healthy and without apparent physical defects [49, 55, 59] in contrast to the embryonic defects observed in *Src*, *Fyn* and *Yes* triple knockout mice [63]. These observations suggest that, in contrast to SFKs, SLAP proteins are not essential for embryonic development. SLAP and SLAP2 functions have been most intensely investigated in hematopoietic tissue and mainly in the context of lymphocyte signaling where they are strongly expressed. These *in vivo* analyses supported a model in which SLAP family members dampen immunoreceptor (TCR and BCR) signaling, thereby influencing lymphocytes development. For instance, disruption of the *Slap1* gene showed that SLAP participates in a novel mechanism of TCR downregulation at the CD4^+^CD8^+^ stage and regulates positive selection. These *in vivo* analyses together with results obtained from cultured cells support a model in which SLAP regulates lymphocytes development by hindering immunoreceptor signaling [48, 64, 65]).

### GRB7 family

The growth factor receptor bound protein-7/10/14 (GRB7/10/14) adaptors were originally identified as partners of activated EGFR [66–68]. Within the GRB7/10/14 family, GRB10 and GRB14 are major negative regulators of insulin and insulin growth factor 1 (IGF1) effects on metabolism and growth [69, 70]. Conversely, GRB7 is implicated in the transduction of FAK- and EPHB1-induced cell migration [71]. Therefore, in this review we will focus on GRB10 and GRB14. These proteins possess several signaling modules, including a poly-Pro region close to their N-terminus, a RAS-associating (RA) domain, followed by a Plekstrin Homology (PH) and a SH2 domain. They also include a region known as BPS (between the PH and SH2 domains) or PIR (phosphorylated insulin receptor-interacting region), which is unique to this adaptor family (Figure 1). Similarly to SLAP, GRB7/10/14 structure and function were acquired relatively late, in evolutionary terms [69]. GRB10 is an imprinted gene that is predominantly expressed from the maternally inherited allele in most human and mouse tissues, except brain [72]. GRB10 and 14 have similar patterns of expression in many tissues, but with some differences. Consistent with their role in insulin signaling, GRB10 and 14 are highly expressed in two major insulin target tissues: skeletal muscle and adipose tissue. GRB10 is also strongly expressed in pancreas, moderately in cardiac muscle and brain, and weakly in many other tissues. GRB14 is highly expressed in heart and liver, and is also detected in pancreas, kidney, gonads, brain and placenta [69]. Although it is unknown whether GRB10 and 14 are transcriptionally regulated by insulin, GRB14 can be up-regulated by insulin [73, 74]. Recently, a couple of studies revealed that mTORC1 directly phosphorylates GRB10, thereby enhancing its stability and, thus, acting as a negative regulator of insulin or IGF-1 signaling [75, 76].

GRB10 and 14 control TK-dependent signaling by a mechanism similar to the one described for SOCS proteins. The BPS domain of GRB10 and 14 specifically interacts with insulin and IGF-1 receptors (IR and IGF-1R) and inhibits their TK activity. Specifically, crystallographic studies showed that the BPS region of GRB14 acts as a pseudo-substrate that binds to the TK domain of IR and inhibits IR catalytic domain [77]. The SH2 domain potentiates the inhibitory effect of the BPS region by binding to the phosphorylated activation loop of the IR catalytic domain, while the RA and PH domains are involved in GRB14 membrane recruitment and participate in IR negative regulation [77, 78]. The interaction between GRB10/14 and IR/IGF-1R can also interfere with downstream partners. For example, GRB10 disrupts the association of insulin receptor substrate (IRS) proteins with IR [79]. In cells expressing IR and PTP1B, GRB14 co-expression maintains the pTyr of the IR activation loop, while favoring dephosphorylation of Tyr972 in the juxtamembrane domain [80]. As Tyr972 is the main docking site for IRS1, this may contribute to GRB14 ability to inhibit the association of IRS1 with IR. GRB10 might regulate the internalization and degradation of its target proteins, such as IGF-1R, by interacting with the ubiquitination factor NEDD4 [81–83]. GRB10 and 14 also regulate signaling initiated by other RTKs [70]. For example, GRB14 inhibits Fibroblasts Growth Factor (FGF) receptor-mediated signaling by altering FGF-induced PLCγ phosphorylation and activation [84]. Mechanistically, GRB14 SH2 binding to FGF receptor induces a conformational change that unmasks a PLCγ binding motif on GRB14, allowing PLCγ trapping and inactivation. On the other hand, GRB14 enhances RET-mediated signaling [85] and GRB10 promotes signaling mediated by PDGFR, vascular endothelial growth factor receptor (VEGFR) and KIT via not well known mechanisms [86–88].

In agreement with their role in insulin signaling, *Grb10* and *Grb14* knockout mice show improved insulin/IGF sensitivity [89–91]. The *Grb10−/−* phenotype also includes embryo and placenta overgrowth as well as increased skeletal muscle and pancreatic β-cell mass [92]. Conversely, transgenic mice overexpressing the *Grb10* maternal allele show postnatal growth retardation, hyperinsulinemia, glucose intolerance and insulin resistance [93]. Accordingly with mouse phenotypes, modulations of GRB10/14 expression in cultured cells show that these adaptors inhibit insulin/IGF-induced receptor activation, revealed by the inhibition of endogenous substrate phosphorylation, such as IRS1, IRS2, SHC and p62DOK, and the inhibition of downstream signaling pathways such as PI3K/AKT and ERK1/2 (reviewed in [69, 70]).

### SH2B family

The SH2B adaptor family includes SH2B1, APS (SH2B2) and LNK (SH2B3). LNK has a well-characterized negative function in JAK2 and TCR signaling [20, 94, 95], while the negative role of the other members remains controversial and may depend on the nature of the TK-dependent signaling. The molecular bases of these specificities are poorly understood, but they may involve alternative splicing of the *SH2B1* and *APS* genes [70, 96]. SH2B family members contain an N-terminal dimerization domain, Pro-rich regions, a central PH domain and an SH2 domain (Figure 1). While highly homologous, the SH2 domains of these adaptors display distinct biochemical characteristics resulting in specific signaling functions. For example, the SH2B1 SH2 domain binds preferentially to JAK2, whereas the APS SH2 domain has higher affinity for the insulin receptor [97]. This specificity is attributable to the difference in the oligomeric states of the two SH2 domains: monomeric for SH2B1 and dimeric for APS [98]. Multiple consensus sites for Tyr and Ser/Thr phosphorylation are also found in SH2B adaptors. The SH2B family is evolutionarily conserved from insects to humans [99, 100]. *Drosophila* has only one dSH2B protein that is structurally similar to SH2B1. The core functions of dSH2B (e.g., growth, reproduction and metabolism) seem to be evolutionarily conserved, but the three mammalian SH2B family members also have new specific functions. Indeed, deletion of SH2B2 or SH2B3 does not alter growth or glucose metabolism in mice, in contrast to SH2B1 knockout [101]. LNK is mainly expressed in hematopoietic tissues, but also in testis, brain and muscle [102]. Similar to other negative regulators, LNK expression is induced following JAK/STAT or Tumor Necrosis Factor (TNF) α signaling, consistent with the existence of a transcription-dependent negative control loop [103, 104].

LNK negatively regulates TCR signaling in a LCK-dependent manner [95] and the activity of cytokine receptors, such as the EPO and thrombopoietin (TPO) receptors, in a JAK2-dependent manner [105, 106]. The role of LNK in TPO signaling has been especially well studied but the molecular mechanism of TPO-mediated signaling inhibition by LNK has not been fully elucidated. Mechanistically, the LNK SH2 domain binds to a JAK2 sequence located within the pseudo-kinase domain linker, upon JAK2 phosphorylation on Tyr813 induced by TPO [107]. The PH domain of LNK is also involved in this regulatory process [108]. As a result, LNK is a potent inhibitor of JAK2/STAT signaling during hematopoiesis. In hematopoietic stem cells, LNK/JAK2 interaction is further negatively regulated by 14-3-3 adaptor proteins. Binding of 14-3-3 to LNK requires phosphorylation of two serine residues in LNK. This binding abrogates LNK/JAK2 interaction, thereby affecting its inhibitory function [109]. LNK also controls signaling through RTKs, including KIT [110, 111], PDGFR [112], FLT3 [113] and CSF1 receptor [114]. For instance, the interaction of LNK SH2 with the phosphorylated Tyr568 present in KIT juxta-membrane domain [115] results in reduction of downstream signaling including the attenuation of GAB2 phosphorylation and MAPK activation [110]. The mechanism underlying this molecular process was not further elucidated in this study. Finally, LNK also interferes with TRKA signaling, possibly by competing with the positive-acting SH2B1 and APS for binding to TRKA [116].

Consistent with LNK negative role in cytokine receptor signaling reported in cell culture, *Lnk*-deficient mice are viable but display abnormal cytokine signaling and aberrations in hematopoiesis [102, 110]. Specifically, these studies revealed defects during lymphopoiesis, erythropoiesis, megakaryopoiesis, mast cell development and macrophages proliferation as well as in hematopoietic stem cell (HSC) expansion (for review, see [117, 118]). Moreover, *Lnk* and *Tpo* double-knockout mice revealed opposite physiological roles for LNK and TPO in HSC expansion [119] and LNK overexpression inhibits megakaryocyte development in mice, consistently with a role in TPO/JAK2 signaling regulation [120]. However, although high levels of LNK are present in non-hematopoietic tissues such as the testis, brain and muscle, no noticeable phenotypes of genetically modified mice were reported in these tissues, questioning about the physiological role of LNK in non-hematopoietic compartments.

### MIG6

Mitogen-inducible gene 6 (MIG6, also known as receptor-associated late transducer, RALT, or ERBB feedback inhibitor 1, ERRFI1) was first cloned from a hydrocortisone-induced rat liver cDNA library [121]. MIG6 was then identified as a partner and an endogenous inhibitor of EGFR (also named ERBB1) and ERBB2, two members of the ERBB receptor family [122, 123]. MIG6 is a cytosolic protein composed of a CDC42/RAC interaction and binding (CRIB) domain, Pro-rich motifs, a 14-3-3 protein binding motif, the RALT endocytic domain (RED) and the ERBB-binding region (EBR) (Figure 1). MIG6 is present in higher order species, suggesting that its expression has been acquired during evolution for regulating more complex signaling circuits [124]. MIG6 is an immediate early response gene that is expressed in various tissues [124]. Its expression can be induced through a RAS/MAPK-dependent mechanism upon sustained stimulation by a broad spectrum of extracellular cues and as such, MIG6 defines a negative regulatory feedback loop that is tightly regulated. Indeed, the transcriptional activation of *ERRFI1* (the gene encoding MIG6) appears to be transient [122] and MIG6 protein is degraded through a proteasome-dependent mechanism upon interaction with DNAJB1 [125, 126].

MIG6 inhibits ERBB kinase activities by direct binding of its EBR region to ERBB catalytic domain. Mechanistically, the N-terminal portion of EBR, called segment 1 (amino acids 336-364), interacts with ERBB C-lobe that overlaps with a site critical for forming the asymmetric dimer with the N-lobe of the other ERBB subunit. As a result, MIG6 locks ERBB in a catalytically inactive conformation and hinders its dimerization required for signal transduction [127]. Recently, it has been shown that phosphorylation on Tyr394 and Tyr395, which are located in segment 2 (amino acids 365-412) of EBR C-terminus, is critical for effective interaction of MIG6 with EGFR [128, 129]. A structural analysis suggests that EBR segment 1 binds across the base of the C lobe of ERBB and segment 2 forms a β-hairpin-like element that occupies the peptide-substrate binding site, once phosphorylated. Interestingly, Tyr394 and Tyr395 are phosphorylated by EGFR and SRC respectively [130], thus creating a forward feedback loop in the control of ERBB activity by MIG6. MIG6 also plays a role in ERBB receptor trafficking. Upon EBR docking onto the receptor, the RED domain interacts with the endocytic proteins AP-2 and intersectins to induce clathrin-mediated ERBB endocytosis [131]. Moreover, MIG6 mediates ERBB receptor sorting to late endosomes by binding to syntaxin 8, thus promoting ERBB lysosomal degradation [132]. MIG6 expression also negatively regulates MET signaling [133]. This activity requires an intact CRIB motif, suggesting that MIG6 acts, at least in part, distally from MET, possibly by inhibiting Rho-like GTPases. Surprisingly, MIG6 can also activate the CTK ABL by interacting with its kinase domain via the EBR domain. MIG6-dependent ABL activation occurs only when ERBB is inactive and leads to the induction of ABL apoptotic function [134]. This sophisticated mechanism is consistent with a switch-like mechanism, whereby MIG6 interacts with and attenuates EGFR activity, while in the absence of ERBB stimulation, it activates ABL pro-apoptotic function.

Gain and loss of function analyses in a wide range of cultured cells show that MIG6 inhibits downstream ERBB signaling, including activation of ERK and AKT, as well as biological responses regulated by ERBB receptors, such as cell proliferation and migration [123, 135–139]. Consistent with these data, *Mig6* gene inactivation in mice induces sustained ERBB signaling, leading to overproliferation and impaired differentiation of epidermal keratinocytes and development of tumors in various tissues [140, 141].

## SMALL ADAPTORS IN THE CONTROL OF ONCOGENIC SIGNALING DRIVEN BY TK IN HUMAN CANCER

**Figure 3:**
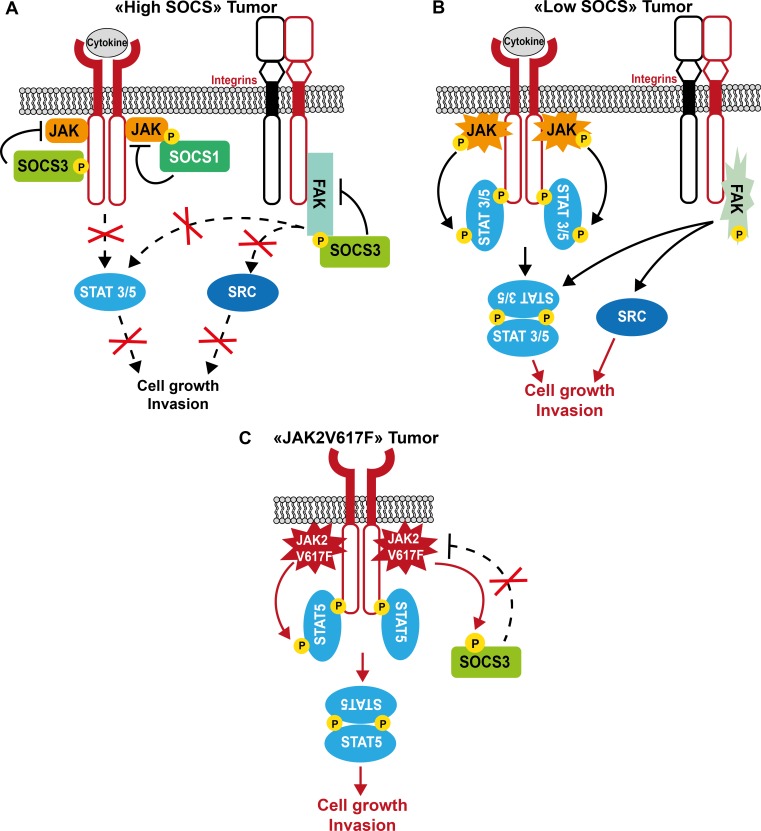
Model of SOCS tumor suppressor function in human cancer **A**. In tumor cells with high SOCS expression, these adaptor proteins inhibit tumor cell growth by controlling JAK/STAT-dependent cytokine signaling, and restrict integrin-dependent cell invasion by inhibiting FAK/SRC signaling. **B**. Upon SOCS inactivation in tumor cells, cytokine and integrin signaling are exacerbated, thus contributing to tumor progression. **C**. Upon expression of the JAK2V617F oncogene, SOCS inhibitory function is inactivated by tyrosine phosphorylation, which results in increased JAK2V617F oncogenic activity.

**Figure 4:**
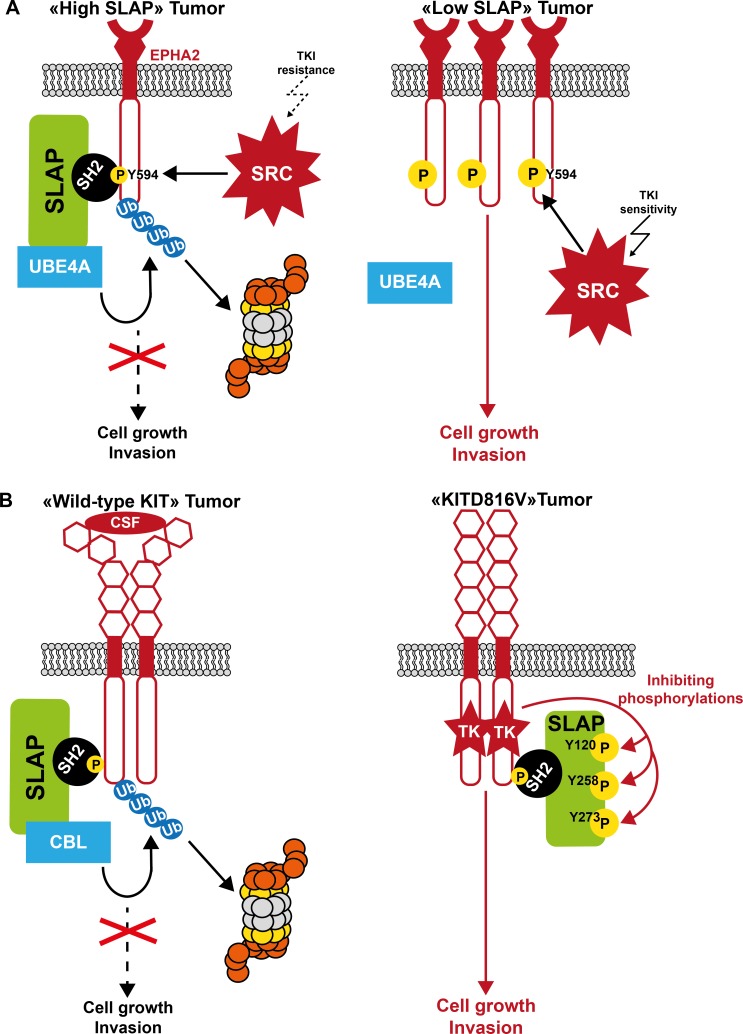
Model of SLAP tumor suppressor function in human cancer **A**. Control of SRC oncogenic signaling by SLAP. In tumor cells with high SLAP expression, SLAP inhibits SRC oncogenic signaling by promoting destabilization of SRC oncogenic substrates, including the cell adhesive receptor EPHA2. This results in the restriction of tumor cell growth and invasion. When SLAP is inactivated, EPHA2 protein level is abnormally increased and SRC oncogenic signaling exacerbated, thus enabling metastatic progression. Consequently, tumor cells may be more sensitive to SRC-like inhibitors. **B**. Control of KIT oncogenic signaling by SLAP. SLAP regulates KIT-driven oncogenic signaling by promoting ubiquitination-dependent KIT degradation. However, upon expression of oncogenic KITD816V, this SLAP-mediated inhibitory mechanism is impaired through tyrosine phosphorylation, thus alleviating SLAP control on KITD816V oncogenic signaling.

**Figure 5:**
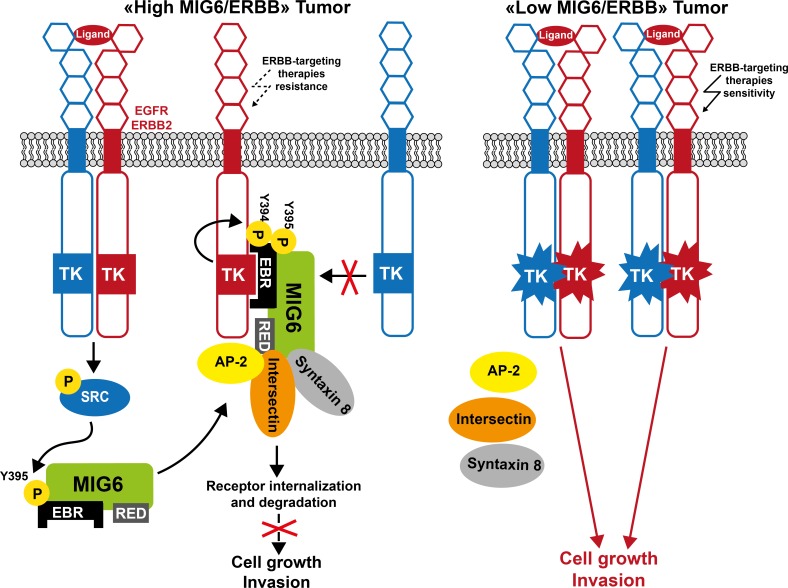
Model of MIG6 tumor suppressor function in human cancer In tumor cells with a high ratio of MIG6/ERBB receptors, MIG6 inhibits the receptor kinase activity and promotes their internalization for lysosomal degradation, resulting in a dramatic reduction of ERBB oncogenic signaling. In tumor cells with a low MIG6/ERBB ratio, MIG6 activity is reduced and ERBB oncogenic signaling is restored. Consequently, tumor cells may be more sensitive to ERBB-like inhibitors.

Most TKs targeted by these adaptor proteins display prominent transforming activity when deregulated. Hence, these negative regulators could also control aberrant signaling driven by oncogenic TKs. As they mostly function by activating a feedback loop, their suppressive activity could be exacerbated by aberrant TK activity in transformed cells. Consequently, they could acquire a potent tumor suppressor function in human cancer. Curiously, their role during malignant cell transformation is much less documented than that of adaptors with positive regulatory functions. In addition, the underlying mechanism of their tumor suppressive role is poorly characterized. Like in normal cells, these adaptors may control TK-dependent oncogenic signaling by directly affecting TK catalytic activity and interaction with components of the signaling cascade, and/or by promoting the destruction of specific elements of this oncogenic signaling, resulting in a restriction of pTyr-dependent cell transformation (Figures 3, 4, 5). Their inactivation by genetic or epigenetic mechanisms could promote TK-driven oncogenic signaling, thus enhancing malignant cell transformation (Table 1). Intriguingly, the tumor suppressive activity of some adaptors appears to be highly context-dependent, because they seem also to participate in the formation of some tumors (Table 1). The molecular cause of these variable effects is unclear. They might target additional processes involved in the control of tumor formation, such as tissue integrity, tumor cell differentiation or tumor immune suppression, resulting in aggravation of the tumor phenotype. A short analysis of the role of these adaptors in human cancer is presented below.

**Table 1:** Status of small adaptors that negatively regulate TK signaling in human cancers

Adaptor	Status in tumors	Type of tumor
SOCS1	Mutation	Lymphoma [144–147], AML [149]
	Hypermethylation	AML [148, 149], CML [150], MPN [151], uterine cervical cancer [152], Barrett's adenocarcinoma [154], ovarian cancer [155], esophageal squamous cell carcinoma [153], glioblastoma multiforme [157], breast cancer [155, 158]
	Hypermethylation/Gene loss	hepatocellular carcinoma [156]
	Down-regulation	Colorectal [159], prostate and pancreatic cancer, myeloma, laryngeal carcinoma [194]
	Up-regulation	*Melanoma* [168]
SOCS2	Hypermethylation	Ovarian cancer [155]
	Hypermethylation/Gene loss	MPN [195, 196]
	Down-regulation	Hepatocellular carcinoma [197], prostate cancer [198, 199]
	Up-regulation	*Acromegaly associated colonic polyps* [200], CML [201, 202], AML, glioblastoma and myeloma [203]
SOCS3	Mutation	MPN [161]
	Hypermethylation	MPN [204], hepatocellular carcinoma [162], glioma [163], cholangiocarcinoma [164], breast [165], lung [205], prostate [206], head and neck cancer [207]; Barrett's adenocarcinoma [154]
	Up-regulation	*Follicular lymphoma* [169]
	Hyperphosphorylation	*MPN* [170]
SOCS4	Hypermethylation	Gastric cancer [208]
SOCS5	Down-regulation	Thyroid gland cancer [209]
SOCS6	Hypermethylation/Gene loss	Gastric cancer [210]
	Gene loss	Colorectal cancer [211]
	Down-regulation	Primary lung squamous cell carcinoma [212], prostate cancer [198], hepatocellular carcinoma [197], liver and thyroid gland cancer [209]
SOCS7	Down-regulation	Breast cancer [158]
SLAP	Down-regulation	Colorectal cancer [52], AML, myeloma [51]
	Up-regulation	CML, chronic lymphocytic leukemia, glioblastoma, prostate cancer [51]
GRB10	Down-regulation	Myeloma, bladder, brain, breast, prostate and pancreatic cancer [75]
	Up-regulation	*Cervical squamous carcinoma* [174], AML [175]
GRB14	Mutation	Colorectal cancer [177]
	Up-regulation	*Thyroid cancer* [85]
LNK	Mutation	MPN, leukemia [117]
	Up-regulation	Skin, kidney, *ovarian* cancer [182]
MIG6	LOH; Gene deletion	Glioblastoma [129, 132, 185]
	Hypermethylation	Papillary thyroid carcinoma [186]
	Down-regulation	Hepatocellular carcinoma [139], breast [183], lung [184], skin, pancreatic and ovarian cancer [140]

Types of tumors where adaptors clearly participate in tumor progression are indicated in italic. LOH, loss of heterozygosity; AML, acute myeloid leukemia; CML, chronic myeloid leukemia; MPN, myleoproliferative neoplasms.

### SOCS family

SOCS proteins regulate inflammation, hematopoiesis, cell growth and metabolism. They might also play important tumor suppressor roles in many cancers [18, 142] where they are frequently inactivated. SOCS1 and 3 anti-oncogenic activities are particularly well documented. For example, SOCS1 can suppress STAT-dependent signaling induced by oncogenic KIT, TEL-JAK2 and BCR-ABL [143]. However, SOCS1 is frequently inactivated in human lymphoma by gene inactivating mutations leading to increased STAT5 and 6 signaling [144–147]. *SOCS1* is commonly silenced by hypermethylation and occasionally mutated in acute myeloid leukemia (AML) [148, 149]. In patients with chronic myeloid leukemia (CML), *SOCS1* is often hypermethylated, but can revert to the unmethylated state during remission [150]. Some BCR-ABL-negative myeloproliferative neoplasms (MPN) also exhibit *SOCS1* hypermethylation, which may complement other mutations, such as the hyperactive JAK2V617F mutation [151]. *SOCS1* hypermethylation is commonly reported in solid tumors [152–155], and combined hypermethylation/gene loss have been observed in hepatocellular carcinoma [156]. However, the functional consequence of SOCS1 inactivation has not been fully elucidated yet. *SOCS1* hypermethylation is associated with enhanced radio-resistance in glioblastoma multiforme, indicative of a pro-apoptotic function [157]. Conversely, higher SOCS1 expression has been observed in early stage tumors and has been linked to better clinical outcome in breast and colorectal cancers (CRC) [158, 159]. A functional analysis suggested that SOCS1 may control CRC metastatic progression, possibly through destabilization of metastatic inducers [159]. SOCS1 is also important for preventing chronic inflammation-mediated carcinogenesis. For instance, *Socs1* knockout mice spontaneously develop intestinal tumors in an IFNγ/STAT1-dependent manner, suggesting that chronic inflammation is a critical determinant for CRC development [160]. Therefore, SOCS1 is a unique anti-oncogene that prevents carcinogenesis by suppressing chronic inflammation.

Although mutations in *SOCS3* are rare events, a loss of function mutation within the SH2 domain of SOCS3 (F136L) was recently described in a cohort of Japanese patients with MPN [161]. *SOCS3* hypermethylation also has been observed in this type of cancer and in solid tumors. For instance, *SOCS3* inactivation increases JAK/STAT and FAK signaling, promoting growth and migration of hepatocellular carcinoma cells [162], invasion of glioma cells [163], enhanced anti-apoptotic IL-6/STAT3 signaling in cholangiocarcinoma cells [164] and, possibly, increased dissemination of breast cancer cells (Figure 3) [165]. *SOCS3* inactivation is also associated with STAT5-dependent CRC metastatic progression [166]. Finally, in mice, SOCS3 can limit inflammation-associated tumorigenesis in colon, by inactivating STAT3 and NFκB [167].

Opposing functions for SOCS proteins have been also reported in some tumors. For example, SOCS1, which is not expressed in normal skin or melanocytic nevi, is up-regulated in melanoma. Moreover, high SOCS1 expression level correlates with the metastatic stage of the disease, suggesting that SOCS1 might be an additional marker of human melanoma progression. Mechanistically, SOCS1 may reduce the tumor cell response to endogenous and/or therapeutically administered cytokines [168]. Similarly, SOCS3 up-regulation was associated with decreased survival in a cohort of patients with de novo follicular lymphoma [169]. Moreover, aberrantly active TKs may find a way to escape SOCS3 negative regulation. For example, JAK2V617F inhibits SOCS3 inhibitory function by promoting SOCS3 hyperphosphorylation in MPN. Additionally, JAK2 diverts SOCS3 to sustain a transformed phenotype by a still not well understood mechanism (Figure 3) [170]. Similarly, truncated G-CSF receptor variants expressed in AML lack the sequences required for SOCS3-mediated control of STAT5 activation [171].

### SLAP family

Little was known about their role in human cancer until recently, mainly because SLAP functions were thought to be restricted to the immune system. Nevertheless, we previously reported that SLAP has a strong capacity to counteract SRC oncogenic activity in fibroblasts through a SH3-dependent mechanism, in agreement with a potential anti-oncogenic activity [58]. More recently, we observed that SLAP is abundantly expressed in colon epithelium, but frequently down-regulated in the associated tumor [52]. SLAP inactivation is not mediated by a gene methylation-dependent mechanism, but in rare cases could be caused by SLAP inactivating mutations located in the SH2 and SH3 domains. SLAP silencing promotes tumor initiation, progression and metastasis formation, while SLAP overexpression inhibits tumor growth and invasion. SLAP promotes the degradation of EPHA2, an important adhesive receptor and key substrate for SRC function in cell tumorigenesis and invasiveness [172, 173]. This novel activity is CBL-independent, but requires SLAP interaction with the ubiquitination factor UBE4A. Mechanistically, SRC phosphorylates EPHA2 on Tyr594, resulting in the promotion of a UBE4A/SLAP/EPHA2 complex for EPHA2 proteasomal degradation, thus limiting the SRC metastatic potential (Figure 4). Consistently, SLAP inactivation in CRC dramatically increases EPHA2 protein level and amplifies the SRC/EPHA2/AKT signaling cascade that promotes tissue invasion of tumor cells. Thus, inactivation of SLAP-mediated degradation of specific SRC substrates defines an additional and important mechanism of SRC-mediated oncogenic induction in CRC. It is not known whether SLAP targets additional SRC oncogenic substrates to mediate its tumor suppressive activity. SLAP expression might exert a similar tumor suppressor function in AML and myeloma [51].

However, deregulated RTKs may evade SLAP negative regulation in pathological conditions. For instance, it was recently reported that wild type KIT, but not the oncogenic KIT-D816V mutant, is degraded through a SLAP-dependent pathway. SLAP can associate with KIT and KIT-D816V, but only KIT-D816V phosphorylates SLAP on Tyr120, Tyr258 and Tyr273, leading to inhibition of SLAP activity and sustained KIT oncogenic signaling (Figure 4) [60].

Finally, SLAP may also participate in cell transformation in specific situations, as reported for SOCS proteins. For instance, SLAP could be involved in the blockade of cell differentiation required for the induction of erythroleukemia by the FLI-1 oncoprotein [62]. Mechanistically, SLAP inhibits several components of EPO receptor signaling needed for late survival and terminal differentiation of erythroblasts. Likewise, SLAP may also be induced in acute promyelocytic leukemia subtypes that harbor FLT3-ITD mutations, suggesting that this adaptor protein might also participate in this transforming process [51].

### GRB7 family

GRB10 and GRB14 roles in human cancer are poorly documented and not clear. GRB10 may participate in progression of some human tumors, as suggested for primary cervical squamous carcinoma [174] and FLT3-ITD positive AML [175], and also in BCR-ABL-mediated leukemogenesis in mice [176]. Similarly GRB14 increases thyroid cancer cell growth by promoting RET signaling and its expression is correlated with human thyroid cancer invasiveness [85]. However, these adaptors may also acquire tumor suppressive functions, as suggested by a recent transcriptomic meta-analysis showing that *GRB10* expression is down-regulated in many human tumors [75]. Moreover, the *GRB14* gene is frequently mutated in human CRC with microsatellite instability [177].

### LNK

LNK may have tumor suppressor function as suggested by the phenotype of *Lnk−/−* mice that resembles the myeloproliferative abnormalities found in human MPN [178]. LNK ectopic expression inhibits proliferation of leukemic cells through binding to and catalytic inhibition of transforming TKs [103, 112, 113, 179–181]. Surprisingly, analysis of LNK expression in a large panel of hematological malignancies revealed that LNK is strongly expressed in nearly half of the patient samples, possibly as a consequence of aberrant cytokine signaling activation [103, 180]. How JAK2 can cope with high LNK level in these cells is unclear. LNK can be mutated in MPN (3-5%) and in some leukemias [117]. Somatic mutations mostly target a hot spot in LNK PH domain, resulting in aberrant JAK/STAT signaling, even in MPN harboring wild type JAK [108]. Consistently, cells co-expressing the TPO receptor and mutated LNK are hyper-responsive to TPO and display aberrant growth and enhanced JAK2/STAT signaling.

Surprisingly, LNK is up-regulated in some solid tumors, including the mesenchymal subtype of serous ovarian cancer, and this is associated with poorer outcome [182]. Functionally, LNK participates in AKT- and MAPK-dependent tumor cell growth and survival.

### MIG6

MIG6 tumor suppressive role is supported by the finding that upon deletion of *ERRFI1*, mice frequently develop spontaneous or chemical-induced tumors [140]. Additionally, MIG6 expression is frequently reduced in various human cancer types [139, 140, 183, 184] and this is correlated with poor survival in patients with breast or lung cancer [183, 184]. MIG6 inactivation can be the result of loss of heterozygosity or focal deletion of *ERRFI1*, as reported in EGFR-amplified glioblastoma [129, 132, 185], or promoter methylation, as found in papillary thyroid carcinoma [186]. MIG6 inactivation may affect RTK aberrant signaling (Figure 5). Accordingly, MIG6 down-regulation in thyroid cancer cells and papillary carcinoma enhances EGFR, ERBB2 and MET activity.

## THERAPEUTIC CONSEQUENCES IN ONCOLOGY

All this data indicates that loss of adaptor-mediated regulation of oncogenic TKs contributes to tumor formation. These novel findings may have significant consequences for cancer treatment/management, such as the discovery/development of novel biomarkers and the improvement of therapy targeting TK-dependent signaling.

### Adaptor expression levels as novel biomarkers

The expression status of some of these adaptors in transformed cells may predict the tumor responses to drug that target oncogenic TK signaling. For instance, low expression in tumor cells of adaptors that control TK catalytic activity, such as MIG6, might predict heightened oncogenic signaling dependent of deregulated TKs, such as ERBB receptors, and therefore may be a good predicator of tumor cell response to TK inhibitors (TKIs) (Figure 5). Indeed, carcinogen-induced tumors in *Errfi1^−/−^* mice are highly sensitive to the EGFR TKI gefitinib [140]. MIG6 silencing also increases bladder cancer cell sensitivity to the therapeutic anti-EGFR antibody cetuximab [187]. A low MIG6/EGFR ratio, predicting high EGFR activity, is highly correlated with erlotinib sensitivity in cancer cell lines derived from different tissues [188]. Analysis of a cohort of patients with lung cancer treated with gefitinib alone demonstrated higher response rates and a marked increase in progression-free survival in patients with a low MIG6/EGFR ratio [188]. Overall, these studies highlight a mechanism of resistance to EGFR-targeted therapies in tumors with high MIG6/EGFR ratio. They also suggest that this ratio could represent a novel biomarker for guiding the decision to incorporate these drugs into chemotherapeutic regimens.

Inactivation in tumor cells of adaptors that control substrate stability, such as SLAP, would also predict heightened oncogenic signaling dependent of the deregulated TK, such as SRC, and therefore may be a good predictor of tumor cell response to TKIs. Conversely, high SLAP expression level might indicate that although SRC activity is aberrant in these tumors, its transforming activity could be hampered by SLAP-mediated destruction of important SRC substrates. This would render tumor cells less dependent on SRC oncogenic signaling and, consequently, rather resistant to a SRC-based therapy (Figure 4). This model has been experimentally verified in CRC cells, where response to SRC inhibitors was greatly enhanced in cells where *SLA* was silenced [52]. Therefore, SLAP down-regulation may be a key predictor of SRC inhibitory response, and may explain, at least in part, why SRC inhibitors failed to generate promising results in solid tumors so far. Overall, these findings support the idea that the level of these regulators may be used as valuable biomarkers to select patients who could respond to TK-targeted therapy.

### Adaptors as potential therapeutic targets

Due to their prominent tumor suppressor function, forced expression of these adaptors in tumor cells could represent an alternative therapeutic strategy to target oncogenic TK signaling in human cancer. Restoration of adaptor expression in tumor cells can reduce tumor growth and/or metastasis formation. Nevertheless, gene therapy is still a technical challenge and alternative methods to modulate adaptor activity or expression might be considered. These adaptor proteins are frequently inactivated in tumors via a methylation-dependent mechanism. Accordingly, demethylation drugs, such as 5-aza-2′-deoxycytidine that exhibits clear tumor suppressive roles, could restore adaptor expression in tumor cells and may represent a potential anti-tumor therapeutic strategy. Forced expression of many of these adaptors, such as LNK [181] and SOCS proteins [18], was used to test their capacity to overcome TK-induced cell transformation. While promising, this strategy may be limited by the capacity of oncogenic TKs to escape negative regulation by phosphorylation-dependent mechanisms, as reported for SOCS and SLAP. Besides, modulation of their expression in tumor cells for anti-tumor therapy is highly context-dependent due to their opposite function in different cancers, as described above. Another potential therapeutic strategy, although not experimentally validated, would arise from the development of small molecules that mimic their actions on their capacity to inhibit TK catalytic activity as demonstrated for the targeting of JAKs by the SOCS3-KIR domain.

Finally, because of their prominent role in immune response, their level of expression may dictate the tumor cell response to cytokine-based therapy. For instance, SOCS1 and SOCS3 up-regulation seem to be responsible for the unresponsiveness to IFN therapy in patients with leukemia. Indeed, SOCS1 constitutive expression has been observed in patients with CML [189], in agreement with the reported hypomethylation of this gene [190], and was correlated with poor response to IFNα treatment, possibly due to a direct effect on receptor signaling [189]. SOCS3 expression is also elevated in CML and confers resistance to IFNα treatment [191]. SOCS1 expression is also higher in IFN-resistant neuroendocrine tumor cells and siRNA inhibition of SOCS1 expression enhances their IFN-responsiveness [192]. Likewise, siRNA-mediated inhibition of SOCS1 and SOCS3 expression in melanoma cells enhances their responsiveness to IFN [193]. This supports the idea that siRNA-mediated reduction of SOCS level could be a promising new approach to enhance IFN therapeutic effectiveness.

## CONCLUDING REMARKS AND PERSPECTIVES

Since the discovery of CIS twenty years ago, this small class of adaptor proteins has emerged as an important player in the mechanisms to finely tune TK-mediated signal transduction. While their functions have been mostly investigated in hematopoietic cells due to their high expression levels and to the essential role of TK signaling in hematopoiesis, more recent data revealed additional important roles in non-haematopoietic cells, as exemplified by the SLAP tumor suppressive role in the colon. Therefore, one important perspective will be to uncover such physio-pathological activities in non-hematopoietic tissues using novel relevant *in vitro* and animal models. Similarly, while underlying mechanisms of adaptor-mediated negative feedback regulation was thought to be rather well established, recent molecular analyses challenged this idea and suggest the existence of broader mechanisms involved in their activities. For example, CBL has been reported as the main SLAP effector in the control of TK signaling through ubiquitin-mediated degradation via the proteasome or the lysosome [45, 53–55] but our recent study revealed that SLAP recruits distinct ubiquitination factors such as UBE4A in the colon to control transforming substrates stability, despite a high expression level of CBL in this tissue [52]. Additionally, proteomics identified a dozen of ubiquitination factors as specific SLAP interactors in CRC suggesting that these adaptors share common mechanisms to control signaling, but recruit a large repertoire of effectors to target a wide range of TK-dependent signaling. Surprisingly, proteomics also identified a hundred of additional SLAP binders unrelated to the control of receptor signaling or protein stability, predicting a much broader role of this adaptor in signal transduction and raising the question whether its functions are restricted to TK-dependent signaling [52]. We thus anticipate novel functions for these adaptors to be identified in the future and proteomic methods combined with functional analyses are warranted to give a comprehensive view on the role of these adaptors in cell biology.

Due to their feedback role in the control of TK signaling, the negative function of these adaptors is expected to be exacerbated upon aberrant expression of TK activities, uncovering novel tumor suppressor functions for these adaptors. This idea has been experimentally validated in myeloproliferative malignancies (e.g. LNK) but tumor suppressor and anti-metastatic activity for these adaptors have been now reported also in solid tumors (e.g. SLAP and SOCS1). Therefore, a broader tumor suppressor role for these adaptors is expected in human cancer, which will deserve more investigation in the future. Besides, we suspect that oncogenic TKs may find a way to overcome this control mechanism and in some situations divert this regulatory process to sustain tumor progression. How oncogenic TKs cope with this control mechanism is an additional important issue to be addressed in the future. Intriguingly, the function of some adaptors in cancer is more complex because their activity seems to be context-dependent. The reason for such variable activity is quite obscure, but we suggest that these molecules target additional important signals that are involved in the control of tumor development/progression, such as the immune response. It will be thus important to elucidate such tumor promoting function in order to better understand their general role in this human disease and improve TK-based therapeutic strategy according to their activities. In conclusion, this rather neglected mechanism in the control of TK signaling is emerging as an additional important mechanism for the control of human malignancy and a better understanding on the role of this adaptor family in human cancer should ultimately improve TK-based tumor therapies.
